# Supplementary material for: Light-Quality-Dependent Greening and Steroidal Glycoalkaloid Accumulation in Potato Tubers: Regulatory Mechanisms and Postharvest Strategies to Reduce Food Safety Risks
Source: Foods. 2025 Sep 30;14(19):3394. doi: 10.3390/foods14193394 (PMC12523430; doi:10.3390/foods14193394)
Supplement: Supplementary file 1 [file foods-14-03394-s001.zip › foods-3885104-supplementary.pdf]

Table S1. Primer sequences used for RT-qPCR.

Gene Name	Upstream primer sequence (5'→3')	Downstream primer sequence (5'→3')
COP1	GGAGTATTAGTGCCGACGGTGAAC	TTCCTCTTCCTCCTCCGCAACTC
GUN4	CTACCACCACCACCACCCCTAC	TCCTCGTTTAACTGCTGCTTCTCC
HY5	TGCCGCTAGTTCACTACCTTCAAG	TCGCTTCTCCGCCCATCTCC
PIF3	GGATGGGATTTGGGATGGGTATGC	TGGAAGCAGGAATAGGAGGTGAGG
CHID	GACGCTAGTGGGAGTATGGCATTG	TCAGCAGCATCTCCTCGGAAGG
CHLI1	ACCAAGAAGTAATGAGCGCCGAAG	TTGTCCCACACACCCTGTCCTC
GAME4	TGAACCTGTTGCTCTTATGTCTGTC	GATGCTCTTCTCTTTACTATTTCCTCTTG
HMGR	TTCGGATAAGAAGCCAGCAGCAG	CTCCACAAGAGCAGCAACCTCAG
SGT1	ACATTCAGCAACAGGCGGGTTC	CCCAATCCCCTAACCTCGACTACC
SGT3	CCAAGGCTGGGCACCACAAC	TGGAACACCTACGGCTAGAGACTC
SQS	ATGGGAACATTGAGGGCGATTCTG	TCCGGCGGGATCTGCTTCTC
SSR2	AAAGGCAGGGTGACACCGAATATG	AGTTGGCGGCATTTCTCTGAACC
Actin	AGGAGCATCCTGTCCTCCTAA	CACCATCACCAGAGTCCAACA

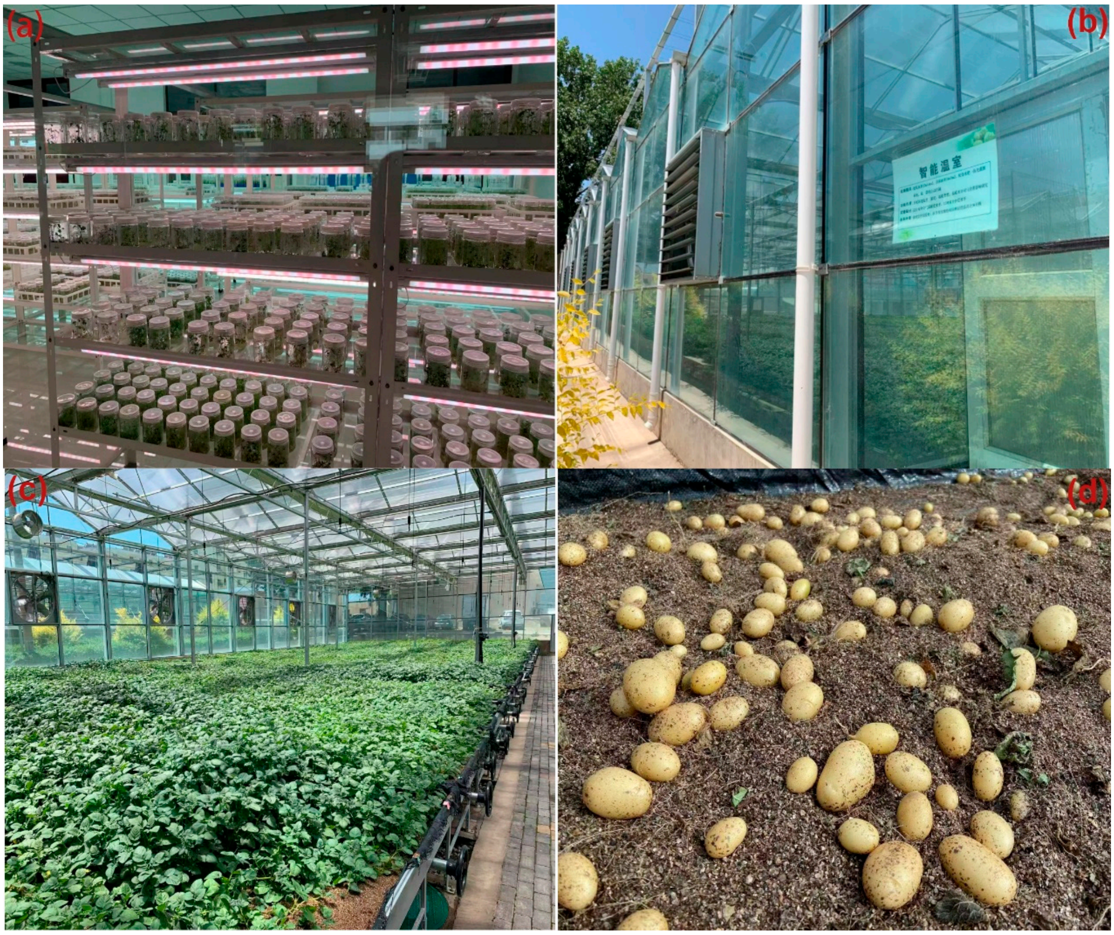

Figure S1. Experimental greenhouse for microtuber cultivation. (a) Sterile tissue culture seedling bottles; (b) intelligent greenhouse; (c) aseptic soilless substrate cultivation; (d) mature mini-tubers ready for harvest.
